# Increase in Enterovirus D68 Infections in Young Children, United Kingdom, 2006–2016

**DOI:** 10.3201/eid2506.181759

**Published:** 2019-06

**Authors:** Everlyn Kamau, Heli Harvala, Soile Blomqvist, Dung Nguyen, Peter Horby, Richard Pebody, Peter Simmonds

**Affiliations:** University of Oxford, Oxford, UK (E. Kamau, D. Nguyen, P. Horby, P. Simmonds); National Health Service Blood and Transplant, London, UK (H. Harvala);; National Institute for Health and Welfare, Helsinki, Finland (S. Blomqvist); Public Health England, London (R. Pebody)

**Keywords:** viruses, epidemiology, respiratory infection, neutralization, seroprevalence, enterovirus, Picornaviridae, United Kingdom

## Abstract

We determined the change in seroprevalence of enterovirus D68 (EV-D68) in the United Kingdom in age-stratified cohorts from 2006 to 2016, the period during which EV-D68 emerged as a cause of severe respiratory disease occasionally leading to paralysis. Infections were acquired primarily in infants and young children, and incidence was markedly higher in 2016.

Enterovirus D68 (EV-D68) is a member of the *Enterovirus D* species (genus *Enterovirus;* family *Picornaviridae*). EV-D68 is distributed worldwide and is typically associated with upper respiratory tract infections. Although EV-D68 infections were infrequently reported in the United States and elsewhere before 2010 (*1*,*2*), multiple novel clades of EV-D68 have emerged worldwide (*3*) and have been associated with occasional outbreaks of more severe respiratory infections (*4*). However, it was not until 2014 that a series of large-scale EV-D68 outbreaks resulting in severe illness and death were reported from the United States and Canada and subsequently, in 2016, from Europe (Netherlands, Spain, Germany, Italy, Ireland, Austria, France, Luxembourg), Japan, China, and elsewhere in Asia (*4*–*6*). 

While primarily regarded as a respiratory pathogen, EV-D68 has been occasionally associated with acute flaccid myelitis (AFM) (*7*). This apparent change in tropism for cells in the central nervous system (*8*) may be linked to the emergence of novel genetically distinct EV-D68 lineages (*3*). An alternative possibility is that the increased number of reports of severe, AFM-associated infections with EV-D68 reflects larger-scale changes in population immunity that have enabled outbreaks to occur in potentially vulnerable age groups. Severe infections typically target infants >6 months of age, when maternal antibody protection wanes (*4*). The World Health Organization recently identified EV-D68 as a potential major public health risk and recommends enhanced surveillance and more effective diagnostics (*9*). We investigated potential changes in exposure to EV-D68 in the general population of the United Kingdom over the period in which the worldwide outbreaks of EV-D68 occurred.

## The Study

**Figure 1:**
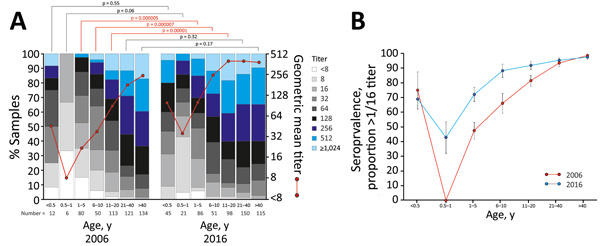
Comparison of enterovirus D68 (EV-D68) seroprevalence in the United Kingdom in 2006 and 2016. A) Seroreactivity to EV-D68 of samples collected in 2006 and 2016 from different age categories. Results are expressed as percentage of samples displaying neutralizing antibody titers <8 or >1,024 (histogram) and geometric mean titers (red line). We performed the Kruskal-Wallace nonparametric test to evaluate differences in titer distributions between samples collected at the 2 points in each band (red text indicates p<0.05). B) Seroprevalence of neutralizing antibodies to EV-D68 in different age categories in 2006 (red) and 2016 (blue). Error bars show SEs of the proportions.

For this study, we obtained serum samples collected as an approximately representative age-stratified cross section of the UK population (*10*). These samples were collected in 2006, before the reports of increased number of EV-D68 cases (n = 516), and in 2016, after the 2014 EV-D68 outbreak (n = 566) (Figure 1, panel A). We used a standard microneutralization assay for serum samples (Appendix).

To determine the optimal strain to measure neutralizing antibody titers (Nab) assays, we compared titer of selected serum samples to the prototype Fermon (1962) strain and those of more recent EV-D68 isolates isolated in 2005 (FI_2005) and 2016 (FI_2016) (Appendix Figure 1). We identified genotypes of D for FI_2005 and B3 for FI_2016 by phylogenetic comparison of the viral protein 1 sequences. We selected samples for comparing titers to narrow down times of EV-D68 exposure: patients >40 years of age in 2006, representing serologic responses to infections acquired substantially before 2006; patients 6 months–5 years of age in 2006, representing responses to infections acquired during 2001–2006; and patients 6 months–5 years of age in 2016, representing responses to infections acquired during 2011–2016. Geometric mean titers (GMTs) of NAbs to Fermon and FI_2016 were comparable between exposure groups (Appendix Figure 2), whereas samples collected from children infected during 2001–2006 showed some evidence for proportionately higher seroreactivity to the 2005 strain. Overall, differences in GMTs were minor, and we selected the FI_2016 strain for NAb screening.

We determined seroreactivity to EV-D68 by GMT calculations for each age and year category and proportions of samples with different neutralizing antibody titers (Figure 1, panel A). We determined seroprevalence and inferred frequencies of past infection using a conservative 1:16 titer threshold (Figure 1, panel B). Frequencies and titers of EV-D68 NAbs differed substantially between the 2 collection years in young children (in the categories 0.5–1 year, 1–5 years, and 6–10 years of age). The difference narrowed in older age groups, and seroprevalence approached 100% in those >40 years of age. Seroprevalence and titer distributions were elevated in the <0.5-year age group, likely reflecting the presence of maternal antibody in these infants.

**Figure 2:**
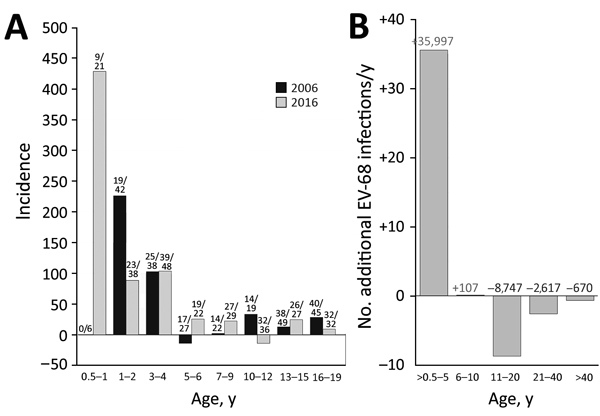
Comparison of incidence of enterovirus D68 (EV-D68) in the United Kingdom in 2006 and 2016. A) Estimated annual incidence of EV-D68 infection for each age group. Incidence was inferred from the difference in seroprevalence from that of the previous age band and converted into infections/year/1,000 population (by dividing the difference in prevalence by the number of years in the age band and multiplying by 1,000). Frequencies of samples with neutralizing antibody titer >16 are shown above bars. B) Change in incidence of EV-D68 infections from 2006 to 2016, expressed as additional EV-68 infections/year (y-axis scale). Figures above bars indicate the predicted positive (red) and negative (blue) change in number infections if these incidences were applied to the whole UK population, based on age-stratified population totals for 2016 obtained from Statista (https://www.statista.com/statistics/281174/uk-population-by-age).

The differences in seroprevalence in the young children between 2006 and 2016 demonstrate greater infection rates in 2016. To identify the age at which this greater exposure occurred, we divided sample sets into narrower age bands and determined seroprevalence (Figure 2, panel A). For both groups, infections were acquired at a very early age, with extremely high incidences in the 0.5–2-year and 3–4-year age ranges in both sample years but a marked reduction for children >5 years of age. Annualized incidence of EV-D68 infection in the 0.5–5-year age band increased from 36 infections/1,000 population during 2001–2005 to 53 infections/1,000 population during 2012–2016, an increase of 50% (Figure 2, panel B). The increased incidence in the <5-year age group in our study would equate to >35,000 additional EV-D68 infections/year, primarily in young children 0.5–2 years of age. Incidence in older age groups was comparable or reduced, as we expected with greater rates of EV-D68 exposure and seroconversion in the younger age ranges.

## Conclusions

The seroprevalence of EV-D68 approached 100% in adult UK populations in this study, consistent with previous seroepidemiology studies conducted in Finland and China (*11*,*12*). Our findings provide further evidence for the wide circulation of EV-D68 infections before reported outbreaks in 2014 and 2016. We demonstrated high incidences of EV-D68 infection in young children; around one half are already infected by 2 years of age in both sampling periods. However, the seroprevalence of EV-D68 infection was consistently higher throughout childhood in 2016 samples than in 2006 samples (Figure 1).

Changes in population immunity, virus antigenicity, transmissibility, cellular tropism, and pathogenicity may contribute to the recent upsurge of severe EV-D68 infections worldwide. Concerning the first potential explanation, seroprevalences in age groups <40 years were consistently lower in 2006 than 2016 (Figure 1, panel B), although EV-D68 circulated extensively, and adults were almost always seropositive and immune before and after periods of greater disease severity in the United Kingdom and elsewhere (*4*,*13*). Changes in NAb susceptibility and escape from population immunity appear unlikely to be a cause of changes in incidence; we found no evidence for major differences in antigenicity between Fermon, genotype D, or B3 isolates (Appendix Figure 1). Another possible cause is increased transmissibility of subgenotypes B2 and B3 in younger age groups (Figure 2, panel A), reflecting a possible change in tropism or persistence of virus shedding and longer periods of infectivity after changes in receptor use, cellular tropisms, or both. The Fermon prototype strain of EV-D68 isolated in 1962 has a stronger affinity to α2,6-sialic acid primarily in the airways than to α2,3-linked sialic acid present in the lower respiratory tract (*14*). Whether the newer EV-D68 strains causing an outbreak of severe respiratory infections in 2008–2010 were evolved to use primarily α2,3-linked sialic acid or have switched to a sialic acid-independent mechanism of virus entry (*14*), such as ICAM5 (*15*), needs further investigation. Of note, only contemporary EV-D68 strains were able to infect neuroblastoma-derived neuronal cell line SH-SY5Y and cause paralysis in a mouse model, indicating that additional changes in virus strains might have occurred around or after 2012 (*8*), although the relationship of this finding to increased transmissibility of EV-D68 has not been studied.

In summary, we document the greater circulation and force of EV-D68 infection in infants and young children in the United Kingdom over the period in which EV-D68 has emerged as an important respiratory pathogen and potential cause of paralytic disease, reflecting its changed transmissibility and pathogenic potential. Such rapid changes in virus behavior emphasize the importance of ongoing surveillance and appropriate diagnostics for emerging enteroviruses in the future.

Appendix Additional information about increased incidence of enterovirus D68 infections in young children between 2006 and 2016.

## References

[1] Khetsuriani N, Lamonte-Fowlkes A, Oberst S, Pallansch MA; Centers for Disease Control and Prevention. Enterovirus surveillance—United States, 1970-2005. MMWR Surveill Summ. 2006;55:1–20. 16971890

[2] Centers for Disease Control and Prevention (CDC). Clusters of acute respiratory illness associated with human enterovirus 68—Asia, Europe, and United States, 2008-2010. MMWR Morb Mortal Wkly Rep. 2011;60:1301–4. 21956405

[3] Tokarz R, Firth C, Madhi SA, Howie SR, Wu W, Sall AA, et al. Worldwide emergence of multiple clades of enterovirus 68. J Gen Virol. 2012;93:1952–8. 10.1099/vir.0.043935-0 22694903 PMC3542132

[4] Holm-Hansen CC, Midgley SE, Fischer TK. Global emergence of enterovirus D68: a systematic review. Lancet Infect Dis. 2016;16:e64–75. 10.1016/S1473-3099(15)00543-5 26929196

[5] Knoester M, Schölvinck EH, Poelman R, Smit S, Vermont CL, Niesters HG, et al. Upsurge of Enterovirus D68, the Netherlands, 2016. Emerg Infect Dis. 2017;23:140–3. 10.3201/eid2301.161313 27660916 PMC5176244

[6] Messacar K, Robinson CC, Pretty K, Yuan J, Dominguez SR. Surveillance for enterovirus D68 in colorado children reveals continued circulation. J Clin Virol. 2017;92:39–41. 10.1016/j.jcv.2017.05.009 28521212 PMC5625344

[7] Sejvar JJ, Lopez AS, Cortese MM, Leshem E, Pastula DM, Miller L, et al. Acute flaccid myelitis in the United States, August–December 2014: results of nationwide surveillance. Clin Infect Dis. 2016;63:737–45. 10.1093/cid/ciw372 27318332 PMC5709818

[8] Brown DM, Hixon AM, Oldfield LM, Zhang Y, Novotny M, Wang W, et al. Contemporary circulating enterovirus D68 strains have acquired the capacity for viral entry and replication in human neuronal cells. MBio. 2018;9:e01954–18. 10.1128/mBio.01954-18 30327438 PMC6191546

[9] World Health Organization. R&D Blueprint: list of Blueprint priority diseases. 2018 [cited 2018 Dec 11]. https://www.who.int/blueprint/priority-diseases

[10] Osborne K, Gay N, Hesketh L, Morgan-Capner P, Miller E. Ten years of serological surveillance in England and Wales: methods, results, implications and action. Int J Epidemiol. 2000;29:362–8. 10.1093/ije/29.2.362 10817137

[11] Smura T, Ylipaasto P, Klemola P, Kaijalainen S, Kyllönen L, Sordi V, et al. Cellular tropism of human enterovirus D species serotypes EV-94, EV-70, and EV-68 in vitro: implications for pathogenesis. J Med Virol. 2010;82:1940–9. 10.1002/jmv.21894 20872722

[12] Sun SY, Gao F, Hu YL, Bian LL, Mao QY, Wu X, et al. Seroepidemiology of enterovirus D68 infection in infants and children in Jiangsu, China. J Infect. 2018;76:563–9. 10.1016/j.jinf.2018.02.003 29428227

[13] Carrion Martin AI, Pebody RG, Danis K, Ellis J, Niazi S, DE Lusignan S, et al. The emergence of enterovirus D68 in England in autumn 2014 and the necessity for reinforcing enterovirus respiratory screening. Epidemiol Infect. 2017;145:1855–64. 10.1017/S0950268817000590 28367789 PMC9203298

[14] Baggen J, Thibaut HJ, Staring J, Jae LT, Liu Y, Guo H, et al. Enterovirus D68 receptor requirements unveiled by haploid genetics. Proc Natl Acad Sci U S A. 2016;113:1399–404. 10.1073/pnas.1524498113 26787879 PMC4747778

[15] Wei W, Guo H, Chang J, Yu Y, Liu G, Zhang N, et al. ICAM-5/telencephalin is a functional entry receptor for enterovirus D68. Cell Host Microbe. 2016;20:631–41. 10.1016/j.chom.2016.09.013 27923705

