# Supplementary material for: Increase in Enterovirus D68 Infections in Young Children, United Kingdom, 2006–2016
Source: Emerg Infect Dis. 2019 Jun;25(6):1200–3. doi: 10.3201/eid2506.181759 (PMC6537723; doi:10.3201/eid2506.181759)
Supplement: Appendix — Additional information about increased incidence of enterovirus D68 infections in young children between 2006 and 2016. [file 18-1759-Techapp-s1.pdf]

Article DOI: https://doi.org/10.3201/eid2506.181759

Increase in Enterovirus D68 Infections in Young Children, United Kingdom, 2006–2016

Appendix

Neutralization Assay

Aliquots of serum samples were inactivated at +56°C for 30 min before assaying for neutralizing activity. Replicate serial 2-fold dilutions of samples were mixed with an equal volume of virus (100 $TCID_{50}$) in a volume of 100 µL to produce a dilution range from 1:8 to 1:1024 and incubated at +37°C for 1 hr. Subsequently, 100 µL of medium containing RD (human epithelial lung) cells was added and incubated at +37°C for 5 days. Cells were then scored for cytopathic effect (CPE) indicating the presence of non-neutralized virus. Each run included the following controls:

a) Toxicity control. A 1/8 dilution of each test sample was incubated with cells and no virus to ensure than any observed CPE in the titration was virally induced.

b) Back-titration of virus infectivity, to demonstrate that ≈100 $TCID_{50}$ has been used in each well.

c) Neutralization susceptibility, titration of virus with the ATCC D68 (ATCC VR-1826) control antiserum, where titers of between 1/2048 and 1/4096 were observed.

Titers were recorded as the highest dilution preventing virus replication. In situations where 1 replicate was positive and 1 was negative, titers were recorded as an intermediate titer (half-log2) dilution. Samples with no CPE at either 1/8 dilution were scored as 1/4; those showing neutralization in both 1/1024 dilutions were scored as 1/2048. For analysis purposes, a conservative threshold of 1/16 was used to assign samples as seropositive and seronegative because samples lacked a clear differentiation into negative and positive titer ranges. The 1/16

titer threshold corresponds to the 1/8 titers reported in (*12*) because the latter did not account for the 1:2 dilution effect after adding virus to the antibody dilution.

Sequencing

Viral RNA was extracted from culture supernatants using QIAamp Viral RNA mini kit (cat. 1020953; QIAGEN, www.qiagen.com). A fragment of VP1 gene (750 bp) was amplified for species and type assignment. Extracted RNA was reverse transcribed using random primers and Superscript III (all, Invitrogen, www.thermofisher.com) and then amplified in PCR reactions using Taq 2X Master Mix (NEB, https://www.neb.com) with primers: 5′-GTNACMTGTTTYATGCARACMAACCT-3′ (Forward) and 5′-AATGCWAATGTMGGNTATGTNACMTG-3′ (Reverse). The resulting sense and antisense sequences were assembled to contigs using Sequencher v5.0 (Gene Codes, https://www.genecodes.com).

Accession Numbers

Assembled complete genome sequences of the FI_2005 and FI_2016 used in the study have been submitted to GenBank and have been assigned accession numbersos. MK216564 and MK216565.

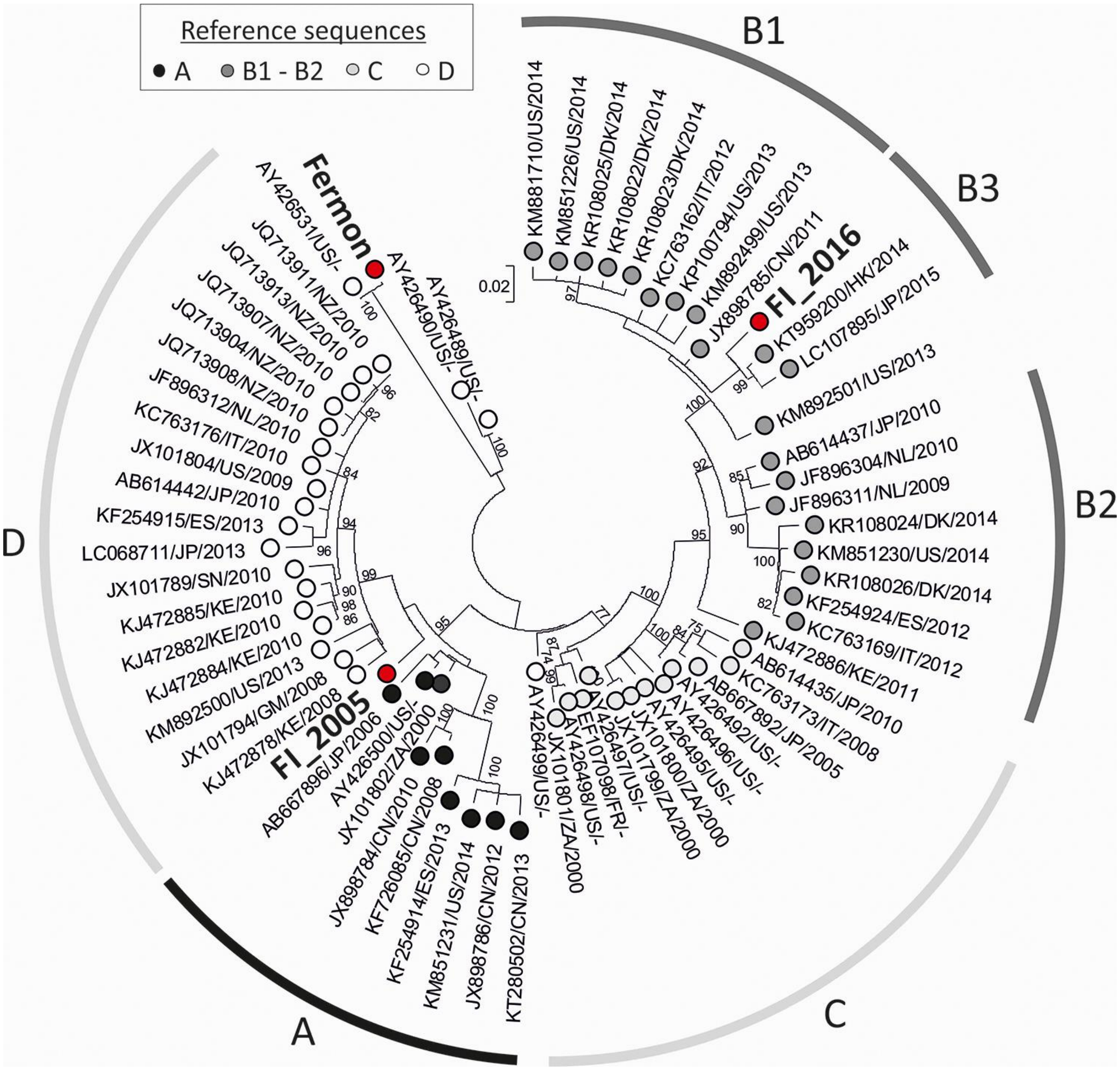

Appendix Figure 1. Phylogenetic analysis of Fermon, FI_2005, and FI_2016 EV-D68 strains used in neutralization assays. Sequences were assembled from Illumina libraries as previously described in Sun et al. (*1*), aligned, and analyzed using SSE platform v1.3 (*2*). Sequences were compared in the VP1 region with published reference sequences; selection was based upon possession of complete VP1 sequences and divergence of <1% from other sequences in this region. Maximum likelihood phylogenetic trees using an optimal substitution model (Tamura 3 parameter model with gamma distribution and allowing for invariant sites as determined by model testing) were constructed using Molecular Evolutionary Genetics Analysis (MEGA) 6 software (*3*). Trees were bootstrap resampled 100 times to assess the robustness of the branches.

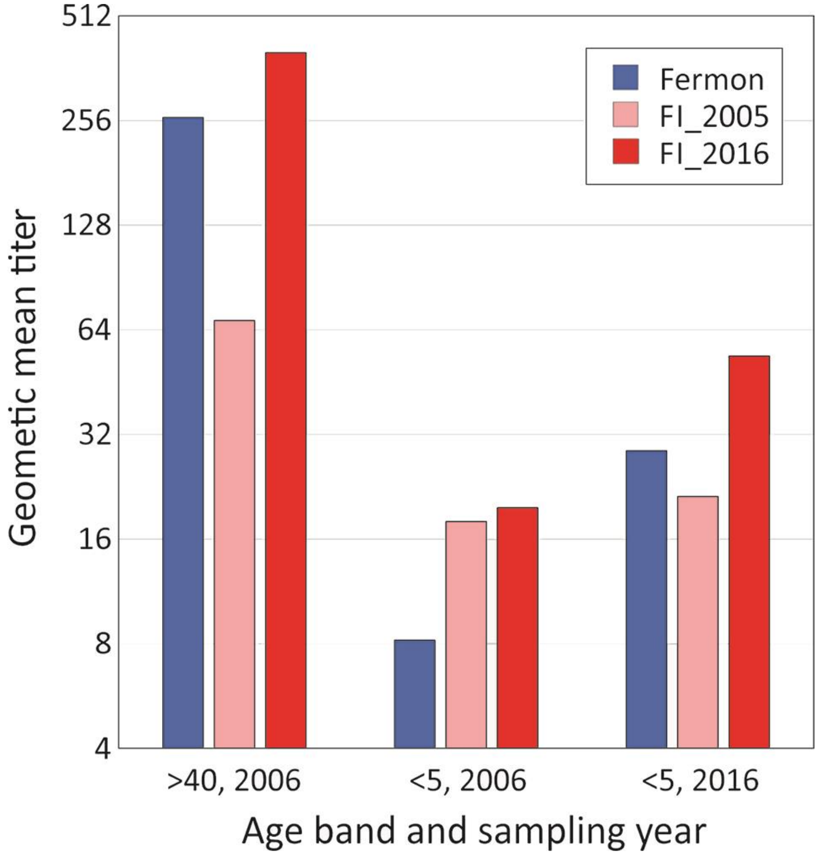

Appendix Figure 2. Geometric mean titers of selected samples from a representative sample of UK residents with 3 strains of enterovirus, representing early (pre-2006), intermediate (2001–2006), and late (2011–2016) exposure. Each group contained 16 samples.

References

1. Thomson E, Ip CL, Badhan A, Christiansen MT, Adamson W, Ansari MA, et al.; STOP-HCV Consortium. Comparison of next-generation sequencing technologies for comprehensive assessment of full-length hepatitis C viral genomes. J Clin Microbiol. 2016;54:2470–84. PubMed https://doi.org/10.1128/JCM.00330-16

2. Simmonds P. SSE: a nucleotide and amino acid sequence analysis platform. BMC Res Notes. 2012;5:50. PubMed https://doi.org/10.1186/1756-0500-5-50

3. Tamura K, Stecher G, Peterson D, Filipski A, Kumar S. MEGA6: molecular evolutionary genetics analysis version 6.0. Mol Biol Evol. 2013;30:2725–9. PubMed https://doi.org/10.1093/molbev/mst197
